# Investigation of the Proliferation, Apoptosis/Necrosis, and Cell Cycle Phases in Several Human Multiple Myeloma Cell Lines. Comparison of *Viscum album* QuFrF Extract with Vincristine in an *In Vitro* Model

**DOI:** 10.1100/tsw.2010.30

**Published:** 2010-02-17

**Authors:** Eva Kovacs

**Affiliations:** Cancer Immunology Research, Rheinparkstr 5, 4127 Birsfelden, Switzerland

**Keywords:** vincristine, Viscum album extract, multiple myeloma, apoptosis, necrosis, cell cycle phases, proliferation

## Abstract

Multiple myeloma is a haematological disorder of malignant plasma cells. Interleukin-6 (IL-6) is a potent growth factor for the proliferation of these cells. Vincristine as a chemotherapeutic agent is used mainly in combination with other chemotherapeutic substances in the treatment of different haematological disorders. *Viscum album* QuFrF (VAQuFrF) extract is an experimental drug that is not used in the treatment in tumour patients. It contains 2000 ng lectin and 10 µg viscotoxin in 10 mg extract. In this study, the effects of VAQuFrF extract were compared with those of vincristine in six human multiple myeloma cell lines (Molp-8, LP-1, RPMI-8226, OPM-2, Colo-677, and KMS-12-BM) using an *in vitro* model. As parameters, the IL-6 production, proliferation, apoptosis/necrosis, and cell cycle phases of the cells were taken. To measure the IL-6 production, apoptosis/necrosis, and cell cycle phases, the substances were tested in dose ranges of 10, 50, and 100 µg/10 cells. To measure the proliferation of the cells, the substances were tested in dose ranges of 1, 5, and 10 µg/10 cells. The profile of the antitumour effects of the two substances is identical. (1) Neither VAQuFrF extract nor vincristine produced IL-6 in any cell line. (2) Both substances inhibited the proliferation of the cells (cytostatic effect), arrested the cell cycle phases, and increased the number of apoptotic/necrotic cells (cytocidal effect). At a dose of 10 µg/10 cells, VAQuFrF more effectively inhibited the proliferation than vincristine (** < 0.01) in the cell lines Molp-8, LP-1, and RPMI-8226. (3) VAQuFrF affected the tumour cells mainly via cytostatic effect. Vincristine had a clear cytocidal effect. These findings indicate that VAQuFrF extract could be a novel drug in the treatment of multiple myeloma.

## INTRODUCTION

Multiple myeloma is a haematological disorder of malignant plasma cells. It is characterised by slow proliferation of these tumour cells, mainly in the bone marrow, by production of large amounts of immunoglobulins and osteolytic lesions. Many cytokines play a role in the growth, progression, and dissemination of this disorder. Interleukin-6 (IL-6) is the most potent factor in the proliferation and/or in survival of the malignant plasma cells[1,2]. This cytokine affects myeloma cells via autocrine and/or paracrine regulation mechanisms.

*Viscum album* (VA) extract from European mistletoe plants has fermented and nonfermented preparations. The fermented preparations are used either alone or in combination with chemo/radiotherapy in the treatment of tumour patients. The *V. album* QuFrF (VAQuFrF) is an aqueous unfermented extract of mistletoe plants. This experimental drug is not used in the treatment of tumour patients. Active components of VA extracts include mistletoe lectins (I, II, III) and viscotoxins; additionally aminoacids, polysaccharides, and lipids.

The results of our experimental investigation with multiple myeloma RPMI-8226 and B-cell lymphoma WSU-1 cell lines have shown that this VAQuFrF has cytostatic and cytocidal effects[3,4]. This means that firstly the proliferation of the tumour cells is inhibited (cytostatic effect) and then afterwards these cells die by apoptosis or necrosis (cytocidal effect). Apoptosis is a physiological process of cellular death; its inhibition can lead to cancer. Proapoptotic drugs are important in tumour therapy. It is known that cell cycle–specific (CCS) drugs blocking the cell cycle phase S or G2/M are more potent in inducing apoptosis than other cycle phase blockers[5]. In a previous study, we found that VAQuFrF extract arrests the cell cycle phase G2/M, leading to apoptosis in B-cell lymphoma WSU-1[4].

Vincristine is a chemotherapeutic agent used mainly in combination with other chemotherapeutic substances in the therapy of multiple myeloma. Vincristine inhibits the proliferation of these tumour cells and, as a CCS blocker[5,6,7], arrests the cell cycle phase G2/M by blocking the mitotic spindle formation[8].

In this study, we tested the effects of a VAQuFrF extract having high lectin and low viscotoxin contents (2000 ng lectin and 10 µg viscotoxin in 10 mg extract), and vincristine in an in vitro model. Our purpose was to compare the effects of VAQuFrF extract with those of vincristine. We used six human multiple myeloma cell lines (Molp-8, LP-1, RPMI-8226, OPM-2, Colo-677, and KMS-12-BM). As parameters, we measured the IL-6 production, proliferation, different cell cycle phases, and apoptosis/necrosis in these tumour cell lines.

## MATERIALS AND METHODS

### Test Substances

VAQuFrF extract was obtained from the Hiscia Institute (Arlesheim, Switzerland). According to the manufacturer, the aqueous unfermented solution of extract (10 mg/ml) contains 2000 ng lectin and 10 µg viscotoxin. Vincristine sulfate salt was obtained from Sigma GmbH (Schnelldorf, Germany; No. 8879).

### Cells and Culture Condition

Human myeloma cell lines Molp-8, LP-1, RPMI-8226, OPM-2, Colo-677, and KMS-12-BM were obtained from DSMZ (Braunschweig, Germany). Four cell lines were derived from blood, the last two (Colo-677 and KMS-12-BM) from lymph node and bone marrow. The cells were cultivated in RPMI-1640 supplemented with 10–20% foetal calf serum, 2 m*M* L-glutamine, and 1% gentamicin in a humidified atmosphere with 5% $CO_2$ at 37°C. The doubling times of tumour cell lines were between 35

and 96 h. For the measurement of the parameters, the cell cultures were used within 4–6 weeks after thawing.

## Treatment of Cells for Analysis

To measure cytokine production, apoptosis/necrosis, and cell cycle phases, the cells were cultured at a density of 0.5–0.7 × $10^6$ cells/ml, except for Colo-677 (0.2 × $10^6$ cells/ml). The cells were divided into two groups. Group I: measurement of the cytokine production and apoptosis/necrosis, group II: measurement of the cell cycle phases. After 24 h, the cells were incubated with VAQuFrF extract or vincristine (doses: 0, 10, 50, or 100 µg/$10^6$ cells/ml). The parameters were measured after 24, 48, or 72 h.

To measure proliferation, the cells were cultured at a density of 0.5–0.7 × $10^5$ cells/100 µl, except for Colo-677 (0.2 × $10^5$ cells/100 µl). After 24 h, the cells were incubated with VAQuFrF extract or vincristine (doses: 0, 1, 5, or 10 µg/$10^5$ cells/100 µl). The parameters were measured after 24, 48, or 72 h.

## Measurement of Cytokine Production

The IL-6 production in the supernatant of the cultured cells was determined by chemiluminescent immunometric assay. The lowest detectable level was 2 pg/ml.

## Measurement of the Proliferation

The proliferation was assessed using cell proliferation reagent WST-1 (Roche, Mannheim, Germany; No. 1644-807). The proliferation rate was measured 1, 2, and 4 h after incubation with the reagents. The upper limit of absorbance was 2.0–2.1.

## Measurement of the Cell Cycle Phases

The cell cycle phases GO/GI, S, and G2/M were assessed using the cycle test plus DNA reagent kit on a flow cytometer (BD, BioSciences, San Jose, CA; No. 340242). Briefly: 5 × $10^5$ cells were incubated at room temperature with trypsin buffer and additionally with trypsin inhibitor + RNAse buffer. The values are expressed in percentage of total viable cell number (100%).

## Measurement of Apoptosis and Necrosis

Apoptosis was measured using Annexin V-FITC (BD Biosciences Pharmingen, San Diego, CA; No. 556-570). Necrosis was measured using propidium iodide (PI). Briefly: 1 × $10^5$ cells were incubated with Annexin V-FITC or PI at room temperature in the dark. Thereafter, the samples were analysed in a flow cytometer. Apoptotic cells: Annexin V-FITC positive and PI negative. Necrotic cells: Annexin V-FITC positive and PI positive. The values are given in percent of total cell number.

## Statistical Analysis

Three to five repeated independent measurements were carried out. For the evaluation of the parameters, the Mann-Whitney U-test was used. The limit of significance was taken as $p < 0.05$.

## RESULTS AND DISCUSSION

Multiple myeloma is at present an aggressive, incurable, systemic disease. Systemic therapies and novel substances alone or in combination with other substances are needed for the treatment of this disorder. Vincristine belongs to the vinca alkaloids, and is used mainly combined with doxorubicin and dexamethasone in the therapy of multiple myeloma. VAQuFrF is an aqueous unfermented extract of mistletoe plants growing in the oak tree. The extract has high lectin and low viscotoxin contents (2000 ng lectin and 10 µg viscotoxin in 10 mg extract). It is an experimental drug that is not used in the treatment of tumour patients. In this study, VAQuFrF and vincristine were tested in dose ranges 10, 50, and 100 $\mu g/10^6$ cells/ml, and 1, 5, and 10 $\mu g/10^5$ cells/100 µl, respectively.

There are two important pathways against tumours: to inhibit the tumour cell proliferation (cytostatic effect) and/or to induce the death of the tumour cells (cytocidal effect).

### Inhibition of Proliferation of Multiple Myeloma Cells (Cytostatic Effect)

The values of the treated samples are expressed as percentages of the untreated samples, and are the average of four or five independent experiments. Significance was assessed vs. untreated samples.

#### *Molp-8, LP-1, and RPMI-8226*

Fig. 1 presents the mean values of the proliferation. Both substances inhibited the proliferation markedly. Comparison of VAQuFrF with vincristine: VAQuFrF at the dose of 10 $\mu g/10^5$ cells was more effective than vincristine ($p < 0.01$) at the same dose in all three cell lines. At a dose of 5 $\mu g/10^5$ cells, the inhibitory effect of VAQuFrF was greater only in Molp-8 ($p < 0.05$).

#### *Colo-677, KMS-12-BM, and OPM-2*

Table 1 presents the mean values ± SEM of the proliferation. Comparison of VAQuFrF with vincristine: the inhibitory effect of VAQuFrF was weaker than that of vincristine in the three cell lines and at each investigated time point. The results of an additional investigation indicate that higher doses increase the effect of VAQuFrF (results are not presented).

The antiproliferative effect of VAQuFrF was dose dependent in the cell lines Molp-8, LP-1, RPMI-8226, Colo-677, and KMS-12-BM; that of vincristine did not show dose dependence in any cell line.

### Investigation of Cell Cycle Phases in Multiple Myeloma Cells

Cell division consists of mitosis (M) and interphase, which divides into phases G1, G2, and S. Nondividing cells are in the stable resting phase, called the G0 phase. The blockade of the cell division leads to an arrest in the different cycle phases. This arrest appears as an accumulation of the tumour cells.

Table 2 presents the range of the values of untreated cells in different cell cycle phases. The values are expressed in percentage of total viable cell number (100%) and are the mean of four independent experiments.

Table 3 presents the mean values and the accumulation of treated cells. For a significant increase or decrease, the percentage of the cell number of treated samples was compared with those of untreated samples.

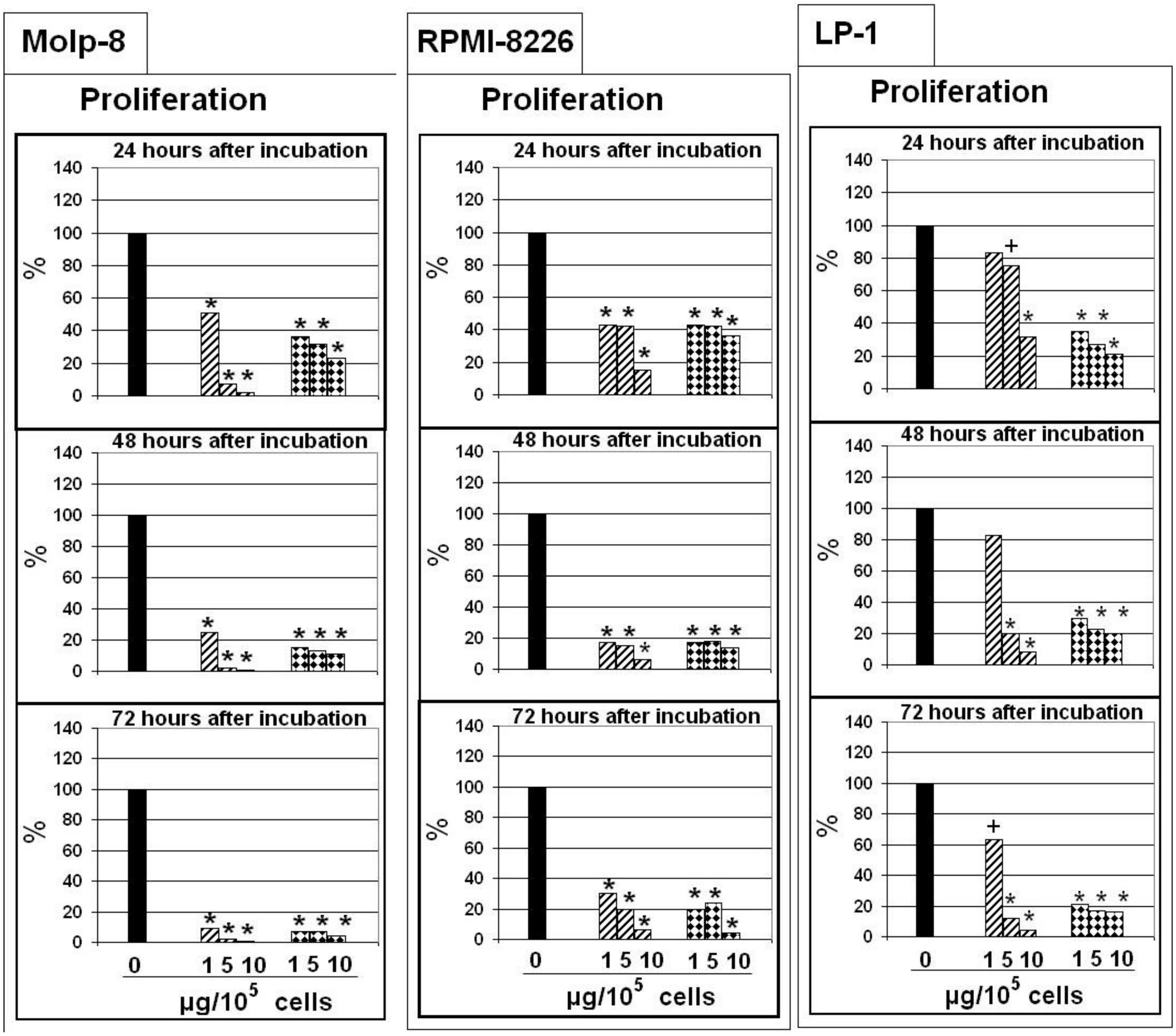

**FIGURE 1.** The effects of VAQuFrF extract (diagonal slash) or vincristine (diamond pattern) on the proliferation of the human multiple myeloma cell lines Molp-8, LP-1, and RPMI-8226. For the proliferation assay, the cells were cultured of a density of 0.5–07 × $10^5$ cells/100 µl. After 24 h, the cells were treated with the test substances for 24, 48, and 72 h. The mean values of four or five independent experiments are expressed as percentage of untreated samples (100%). +$p < 0.05$, *$p < 0.01$ compared with untreated samples (Mann-Whitney U-test).

Phases GO/G1: VAQuFrF led to an accumulation of cells in cell line Molp-8 and LP-1, and in KMS-12-BM ($p < 0.05$ and $p < 0.01$). Vincristine had effect in the cell lines Molp-8 and LP-1 ($p < 0.05$).

Phase S: VAQuFrF increased the cell number ($p < 0.01$) in RPMI-8226. Vincristine had a clear effect for RPMI-8226 and OPM-2 ($p < 0.01$ and $p < 0.05$).

Phases G2/M: VAQuFrF was effective in Colo-677 ($p < 0.01$). Vincristine led to marked increase of the cell numbers in cell lines RPMI-8226, Colo-677, and KMS-12-BM ($p < 0.01$).

It has been reported that vincristine binds to tubulin (membrane protein). By this binding, the mitotic process is blocked, leading to an arrest of the cycle phase in G2/M[5,7]. In this investigation, vincristine led to an accumulation of the cells in cycle phase G2/M in only three out of six multiple myeloma cell lines. This finding is unexpected. It is possible that different tumour cell lines from the same disorder (multiple myeloma) show a different sensitivity to vincristine. The cell number in S and in G0/G1 phases of two cell lines was increased (Table 3), suggesting that vincristine also affects these cycle phases.

**TABLE 1**
**Effects of VAQuFrF and Vincristine on the Proliferation of the Human Multiple Myeloma Cell Lines Colo-677, KMS-12-BM, and OPM-2**

| | VAQuFrF | | | Vincristine | | |
|---|---|---|---|---|---|---|
| | 24 h | 48 h | 72 h | 24 h | 48 h | 72 h |
| Colo-677 | | | | | | |
| 1 µg | 91 ± 6 | 80 ± 4 | 80 ± 8 | 72 ± 11 | 28 ± 10* | 29 ± 9** |
| 5 µg | 74 ± 8 | 47 ± 3* | 52 ± 5* | 68 ± 12* | 24 ± 8** | 27 ± 5** |
| 10 µg | 50 ± 4** | 29 ± 7** | 26 ± 5** | 66 ± 9* | 19 ± 4** | 21 ± 3** |
| KMS-12-BM | | | | | | |
| 1 µg | 98 ± 7 | 93 ± 8 | 104 ± 9 | 19 ± 6** | 3 ± 2** | 4 ± 2** |
| 5 µg | 90 ± 4 | 93 ± 4 | 79 ± 5 | 13 ± 2** | 7 ± 5** | 7 ± 3** |
| 10 µg | 79 ± 5 | 64 ± 6* | 44 ± 4** | 13 ± 2** | 9 ± 3** | 12 ± 1** |
| OPM-2 | | | | | | |
| 1 µg | 98 ± 4 | 97 ± 7 | 100 ± 2 | 15 ± 6** | 17 ± 2** | 19 ± 5** |
| 5 µg | 75 ± 3 | 80 ± 10 | 82 ± 8 | 20 ± 2** | 18 ± 3** | 17 ± 4** |
| 10 µg | 38 ± 9* | 35 ± 9* | 43 ± 4** | 23 ± 2** | 17 ± 3** | 15 ± 2** |

The cells were incubated for 24, 48, and 72 h with VAQuFrF or vincristine (1, 5, and 10 µg/$10^5$ cells). The proliferation of untreated cells was taken as 100%. The values (in %) are the mean ± SEM of three to five independent experiments. *$p < 0.05$; **$p < 0.01$ vs. controls.

**TABLE 2**
**Values of Untreated Human Multiple Myeloma Cells in Different Cell Cycle Phases**

| | G0/G1 | S | G2/M |
|---|---|---|---|
| Molp-8 | 41–52 | 26–44 | 17–23 |
| LP-1 | 50–63 | 27–37 | 8–12 |
| RPMI-8226 | 60–78 | 13–28 | 8–15 |
| Colo-677 | 36–49 | 42–50 | 10–21 |
| KMS-12-BM | 45–53 | 39–48 | 3–10 |
| OPM-2 | 51–62 | 20–38 | 10–19 |

Values are expressed in percentage of total viable cell number (100%). Range of four independent experiments.

The effect of VAQuFrF extract was similar to that of vincristine in that it affected the same cycle phases in four out of six tumour cell lines; however, in a higher dose range. Harmsma et al.[8] described that VAQu extract (a fermented preparation) led to accumulation of cells from lung-colon-breast adenocarcinoma cell lines in S- and G2/M phases. The present results show that VAQuFrF blocks the cells in these cycle phases only in RPMI-8226 and Colo-677, respectively.

The inhibition of the G0/G1 phases in different malignancies shows a correlation with antiproliferative substances[9,10]. In this study, vincristine and VAQuFrF blocked the cells in the G0/G1 phases in the cell lines Molp-8 and LP-1, and also inhibited the cell proliferation markedly.

**TABLE 3**
**Accumulation of Human Multiple Myeloma Cells in Different Cell Cycle Phases**

| | **G0/G1** | | | | | | **S** | | | | | | **G2/M** | | | | | |
|---|---|---|---|---|---|---|---|---|---|---|---|---|---|---|---|---|---|---|
| | **VAQuFrF** | | | **Vincristine** | | | **VAQuFrF** | | | **Vincristine** | | | **VAQuFrF** | | | **Vincristine** | | |
| | **10** | **50** | **100** | **10** | **50** | **100** | **10** | **50** | **100** | **10** | **50** | **100** | **10** | **50** | **100** | **10** | **50** | **100** |
| Molp-8 | | ↑ 46 | ↑ 65 | ↑ 51 | ↑ 59 | ↑ 64 | No effect | | | No effect | | | No effect | | | No effect | | |
| LP-1 | | ↑ 68 | ↑ 72 | | ↑ 78 | ↑ 70 | No effect | | | No effect | | | No effect | | | No effect | | |
| RPMI-8226 | No effect | | | No effect | | | | ↑ 25 | ↑ 33 | ↑ 20 | ↑ 42 | ↑ 45 | No effect | | | ↑ 49 | ↑ 52 | ↑ 50 |
| Colo-677 | No effect | | | No effect | | | No effect | | | No effect | | | | ↑ 18 | ↑ 22 | ↑ 54 | ↑ 62 | ↑ 68 |
| KMS-12-BM | | | ↑ 54 | No effect | | | No effect | | | No effect | | | No effect | | | ↑ 44 | ↑ 56 | ↑ 70 |
| OPM-2 | No effect | | | No effect | | | No effect | | | ↑ 30 | ↑ 31 | ↑ 39 | No effect | | | No effect | | |

Treatment with VAQuFrF or vincristine (10, 50, and 100 µg/$10^6$ cells). Evaluation of four independent experiments. ↑ = accumulation. The numbers present the mean values in percentage.

## Induction of Apoptosis and Necrosis in Multiple Myeloma Cells (Cytocidal Effect)

Cells are born by mitosis and end their life by apoptosis. Both are physiological and active processes. Cancer can occur when the balance between mitosis and apoptosis is disturbed. By apoptosis, unwanted or useless cells are eliminated during development and other normal biological processes. Necrosis is a pathological process that occurs when cells are exposed to a serious physical or chemical insult. It is known that in the late phase of apoptosis, a secondary necrosis occurs.

Fig. 2 presents the mean values of three or four independent experiments, expressed as percentage of total cell numbers. In the untreated samples, the percentage of apoptotic cells lay in the range of 5–38% and that of necrotic cells 10–35%, during 72 h. There were big differences between the tumour cell lines.

### *LP-1, RPMI-8226, Colo-677, and OPM-2*

In these untreated tumour cells, the values of apoptosis lay either in the range of necrosis or below them. VAQuFrF did not greatly alter the apoptosis during the investigation time in any cell line. There was a necrotic effect with a dose dependence (from 50 up to 100 µg/$10^6$ cells) in the cell lines LP-1 and Colo-677 ($p < 0.05$). Vincristine increased the number of apoptotic cells only in LP-1 and Colo-677 ($p < 0.05$); however, without dose dependence. In all four cell lines, the number of necrotic cells was higher than that of apoptotic cells at each dose and at each time point ($p < 0.05$ and $p < 0.01$).

### *Molp-8 and KMS-12-BM*

In these untreated tumour cells, the values of apoptosis lay either in the range of necrosis or above them. Because the proliferation of Molp-8 cells was reduced markedly with VAQuFrF and vincristine, the cells were treated with lower doses (5 and 10 µg/$10^6$ cells). VAQuFrF increased the apoptosis and necrosis in Molp-8 at this dose range ($p < 0.05$ and $p < 0.01$). There was no effect in KMS-12-BM. Vinristine had the same effect on the apoptosis and necrosis in Molp-8 as VAQuFrF. Vincristine was also effective in KMS-12-BM.

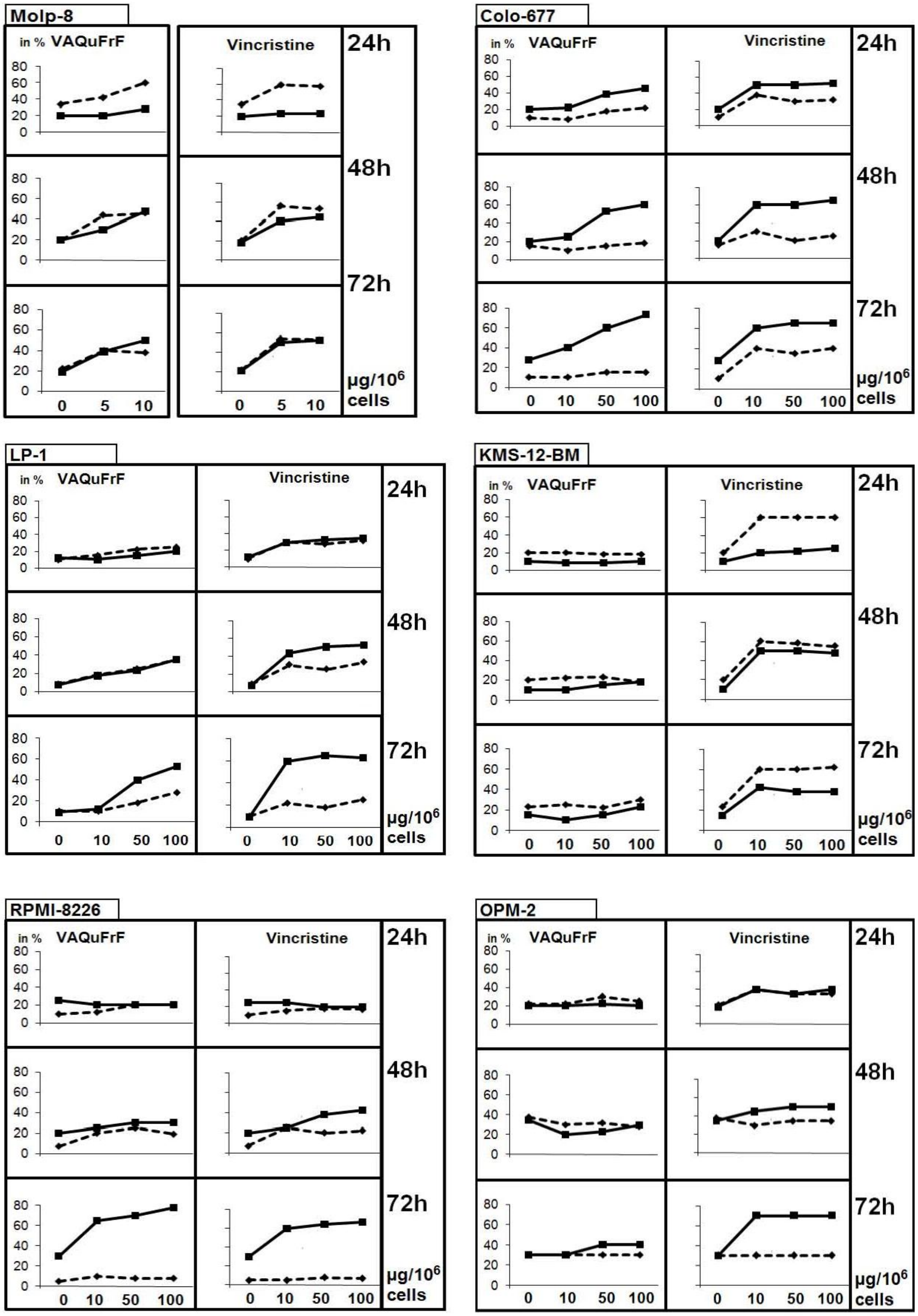

**FIGURE 2**. Apoptosis and necrosis of the human multiple myeloma cell lines Molp-8, LP-1, RPMI-8226, Colo-677, KMS-12-BM, and OPM-2. The cells were incubated with VAQuFrF or vincristine (0, 5, 10, 50, and 100 μg/$10^6$ cells/ml) for 24, 48, and 72 h. The apoptosis was measured using Annexin V-FITC, necrosis using propidium iodide; ♦- - -♦ apoptotic cells, ■—■ necrotic cells. The values are the mean of three or four independent experiments, expressed as percentage of total cell number.

Leoncini et al.[11] found a quantitative correlation between the inhibition of proliferation and apoptosis in lymphoma cells. Stokke et al.[12] reported that inhibition of cell proliferation is a stronger prognostic indicator than the apoptosis in B-cell non-Hodgkin's lymphoma cells.

In this study, the apoptotic/necrotic effect of vincristine was more marked than its proliferative effect in all cell lines. There was no dose dependence between 10, 50, or 100 µg/$10^6$ cells/ml in both parameters. It is possible that vincristine impairs the proliferation and apoptosis/necrosis with a dose dependence only at a lower dose range.

VAQuFrF first inhibits the proliferation and then the cells die by apoptosis and/or necrosis in the multiple myeloma cell lines LP-1, RPMI-8226, and Colo-677, confirming the findings with RPMI-8226 presented in a previous study[3]. Vincristine exerts its apoptotic effect in the S phase[7]. In this investigation, vincristine blocked the cells of OPM-2 and RPMI-8226 in cycle phase S, without influencing apoptosis. The same was found for VAQuFrF in the cell line RPMI-8226.

Chemotherapeutic agents influence apoptosis through a mitocondrial pathway[13]. Multiple myeloma cells overexpress Bcl-2, a mitochondrial membrane protein that suppresses apoptosis[14,15]. Vincristine induces apoptosis via decrease of Bcl-2 in medulloblastoma cells, normal epithelial cells, and fibroblast cells[16]. VAQuFrF decreased the levels of Bcl-2 in B and T lymphocytes[17].

There are several morphological and biochemical differences between apoptosis and necrosis; however, it is difficult to separate the two mechanisms. For anticancer drugs, it is not important which mechanism leads to the death of the tumour cells. At present, it is a "passing fashion" to influence the apoptosis of tumour cells. Don't forget: apoptosis is a physiological process in the life of healthy cells, whereas necrosis is a pathological process for tumour cells.

## Production of IL-6 in Multiple Myeloma Cells

None of the six multiple myeloma cell lines produced IL-6 spontaneously. Treatment with VAQuFrF extract or vincristine also did not lead to IL-6 production (results not shown).

IL-6 is a potent factor in the proliferation and survival of multiple myeloma cells, leading to marked proliferation and the inhibition of apoptosis. IL-6 mediates its effect through the membrane receptors IL-6Rα and IL-6Rβ. IL-6 affects the myeloma cells via autocrine and/or paracrine regulation mechanisms. In the case of autocrine regulation mechanisms, the cytokine is produced endogenously and affects its membrane receptor directly. In the case of paracrine regulation mechanisms, the exogenous cytokine also affects the membrane receptor. In this study, the proliferation was inhibited and the apoptosis/necrosis was promoted by the two test substances. Therefore, endogenous IL-6 production was probably not affected by VAQuFrF and vincristine.

## SUMMARY/CONCLUSION

It was reported previously that VAQuFrF extract and vincristine affect the proliferation, apoptosis/necrosis, and cell cycle phases of the B-cell lymphoma cell line WSU-1. In this study, we compared VAQuFrF extract and vincristine in six human multiple myeloma cell lines using the same parameters. The evaluation shows that the profile of the antitumour effects of the two substances is identical: treatment with VAQuFrF extract or vincristine did not lead to IL-6 production in any cell line. Both substances inhibited the proliferation of the cells (cytostatic effect), arrested cell cycle phases, and increased the number of apoptotic/necrotic cells (cytocidal effect). However, the two substances have different modes of action against tumours: VAQuFrF affects the tumour cells mainly via inhibition of the proliferation, vincristine has a clear cytocidal effect. The findings indicate that VAQuFrF extract could be a novel drug in the therapy of multiple myeloma. The experimental data are reported here for the first time.

## ACKNOWLEDGEMENTS

The author thanks Dr. H. Langemann for the grammatical correction of this manuscript. The measurements of the parameters were carried out in the laboratory of the Society of Cancer Research (Arlesheim Switzerland). The idea of this study is based on the findings of the author and as principal investigator, she wrote the study protocol and coordinated the study. The evaluation of the results, the writing, and the completion of this manuscript were not supported from the Society of Cancer Research or from any foundation.

## References

1. Kawano, M., Hirano, T., Matsuda, T., Taga, T., Horii, Y., Iwato, W., Asaoku, H., Tang, B., Tanabe, O., Tanaka, H., Kuramoto, A., and Kishimoto, T. (1988) Autocrine generation and essential requirement of BSF-2/IL-6 for human multiple myeloma. *Nature* **332,** 83–85.
2. Klein, B., Zhang, X.G., Jourdan, M., Content, J., Houssiau, E., Aarden, L., Piechaczyk, M., and Bataille, R. (1989) Paracrine rather than autocrine regulation of myeloma-cell growth and differentation by interleukin-6. *Blood* **73,** 517–526.
3. Kovacs, E., Link, S., and Toffol-Schmidt, U. (2006) Cytostatic and cytocidal effects of mistletoe (Viscum album L.) Quercus extract Iscador. *Drug Res*. **56,** 467–473.
4. Kovacs, E., Link, S., and Toffol-Schmidt, U. (2008) Comparison of Viscum album QuFrF extract with Vincristine in an in vitro model of human B cell lymphoma WSU-1. *Drug Res*. **58,** 592–597.
5. Lin, Ch.-K.E., Nguyen, T.T., Morgan, T.L., Mei, R.L., Kaptein, J.S., Kalunta, C.I., Yen, C.F., Park, E., Zou, H.Y., and Lad, P.M. (1998) Apoptosis may be either suppressed or enhanced with strategic combinations of anti-neoplastic drugs or anti-IgM. *Exp. Cell Res*. **244,** 1–13.
6. Mastberger, S.C., Duivenvoorden, I., Versteegh, R.T., and Geldof, A.A. (2000) Cell cycle arrest and clonogenic tumour cell kill by divergent chemotherapeutic drugs. *Anticancer Res*. **20,** 1833–1838.
7. E1 Alaoui, S., Lawry, J., and Griffan, M. (1997) The cell cycle and induction of apoptosis in a hamster fibrosarcoma cell line treated with anti-cancer drugs: its importance to solid tumour chemotherapy. *J. Neurol*. **31,** 195–207.
8. Harmsma, M., Gromme, M., Ummelen, M., Dignef, W., Tusenius, K.J., and Ramaekers, F.C.S. (2004) Differential effects of Viscum album extract Iscador Qu on cell cycle progression and apoptosis in cancer cells. *Int. J. Oncol*. **125,** 1521–1529.
9. Pellizzaro, C., Speranza, A., Zorzet, S., Crucil, I., Sava, G., Scarlata, I., Cantoni, S., Fedeli, M., and Coradini, D. (2008) Inhibition of human pancreatic cell line MIA PaCa2 proliferation byHA-But, a hyaluronic butyric ester: a preliminary report. *Pancreas* **36,** 15–23.
10. El-Sherbiny, Y.M., Cox, M.C., Ismail, C.A., Shamsuddin, A.M., and Vucenik, I. (2001) G0/G1 arrest and S phase inhibition of human cancer cell lines by inositol hexaphosphate (IP6). *Anticancer Res.* **21,** 2393–2403.
11. Leoncini, L., Vecchio, M.T.D., Megha, T., Barbini, P., Galieni, P., and Pileri, S. (1993) Correlation between apoptotic and proliferative indices in malignant non-Hodgkin's lymphoma. *Am. J. Pathol*. **142,** 755–762.
12. Stokke, T., Holte, H., Smedshammer, L., Smeland, E.B., Kaalhus, O., and Steen, H.B. (1998) Proliferation and apoptosis in malignant and normal cells in B-cell non-Hodgkin's lymphomas. *Br. J. Cancer* **77,** 1832–1838.
13. Oancea, M., Mani, A., Hussein, M.A., and Almasan, A. (2004) Apoptosis of multiple myeloma. *Int. J. Hematol*. **8**, 224–231.
14. Chanen-Khan, A.A. (2004) Bcl-2 antisense therapy in multiple myeloma. *Oncology* **18(13 Suppl. 10),** 21–24.
15. Tsujimoto, Y. and Shimizu, S. (2007) Role of mitochondrial membrane permeability transition in cell death. *Apoptosis* **12,** 835–840.
16. Shinwari, Z., Manogaran, P.S., Alrokayan, S.A., Al-Hussein, K.A., and Aboussekhra, A. (2007) Vincristine and Iomustine induce apoptosis and p21 (WAF1) up-regulation in medulloblastoma and normal human epithelial and fibroblast cells. *Neurooncology* **87,** 123–132.
17. Duong Van Huyen, J.P., Sooryanarayana Delignat, S., Bloch, M.F., Kacatchkine, M.D., and Kaveri, S.V. (2001) Variable sensitivity of lymphoblastoid cells to apoptosis induced by Viscum album QuFrF, a therapeutic preparation of mistletoe lectin. *Chemotherapy* **47,** 366–376.
